# Exposure of Petrol Station Attendants and Auto Mechanics to Premium Motor Sprit Fumes in Calabar, Nigeria

**DOI:** 10.1155/2009/281876

**Published:** 2009-06-23

**Authors:** N. E. Udonwa, E. K. Uko, B. M. Ikpeme, I. A. Ibanga, B. O. Okon

**Affiliations:** ^1^Department of Family Medicine, University of Calabar Teaching Hospital, Calabar 1278, Cross River State, Nigeria; ^2^Department of Haematology and Blood Transfusion, University of Calabar Teaching Hospital, Calabar 1278, Cross River State, Nigeria; ^3^Department of Community Medicine, University of Calabar Teaching Hospital, Calabar 1278, Cross River State, Nigeria

## Abstract

A population-based-cross-sectional survey was carried out to investigate the potential risk of exposure to premium motor spirit (PMS) fumes in Calabar, Nigeria, among Automobile Mechanics (AM), Petrol Station Attendants (PSA) and the general population. Structured questionnaire was administered on the randomly chosen subjects to elicit information on their exposure to PMS. Duration of exposure was taken as the length of work in their various occupations. Venous blood was taken for methaemoglobin (MetHb) and packed cells volume (PCV). Mean MetHb value was higher in AM (7.3%) and PSA (5.8%) than in the subjects from the general population (2.7%). PCV was lower in PSA (30.8%), than AM (33.3%) and the subjects from the general population (40.8%). MetHb level was directly proportional, and PCV inversely related, to the duration of exposure. The study suggested increased exposure to petrol fumes among AM, PSA, and MetHb as a useful biomarker in determining the level of exposure to benzene in petrol vapour.

## 1. Introduction

No alternative to petrol (premium motor spirit (PMS)) has been introduced into the Nigerian automobile industry. Therefore, millions of automobiles on Nigerian roads run on PMS or diesel fuel. PMS contains volatile organic compound (VOC) such as benzene which is limited by regulation to 6–8% of the content of PMS in Nigeria [1] and between 1% (v/v) to 5% in the USA and Europe [2–6]. Nitrobenzene is largely used in the manufacture of aniline and for the production of lubricating oils used in automobiles and machineries among other uses. Few studies have reported haematological disorders associated with the exposure to benzene in the environment in less developed countries [7]. However, there is evidence about health effects linked with low dose exposures to volatile organics including benzene in PMS [8–12]. It is acutely toxic by inhalation, causing mucous membrane irritation, neurological and other symptoms due to respiratory failure. Chronic exposure has been reported to result in bone marrow depression, aplasia and leukaemia, cardiac abnormalities, heart attack, and other cancers of the lung, brain and stomach [13]. Following inhalation, benzene vapour is rapidly absorbed into the blood and distributed throughout the body [13, 14]. One of the effects of benzene in the body is the production of methaemoglobin (MetHb) which differs from haemoglobin. Haemoglobin accepts and transports oxygen only when the iron atom is in its ferrous form. When haemoglobin becomes oxidized, iron is converted to the ferric state (Fe^3+^) or MetHb. MetHb lacks the electron that is needed to form a bond with oxygen and, thus, is incapable of oxygen transport. Since red blood cells are continuously exposed to various oxidant stresses, blood normally contains MetHb levels of between 1 and 3% [15]. Considering that MetHb has little affinity for oxygen, transport of the later to cells is hindered resulting in functional anaemia [15, 16]. As the level of MetHb increases, symptoms like shortness of breath, palpitation, anxiety and confusion occur [15, 16]. Humans are commonly exposed to benzene and other VOCs through the pulmonary and dermal pathways.

Exposure assessment studies have indicated that important microenvironments for benzene exposure are those associated with petrol use: driving, working at or visiting a service station, having an attachment to an automobile mechanic (AM) workshop, and living close to waste sites of petroleum refinery or chemical manufacturing plants [2]. Benzene uptake and distribution have been investigated relying on unmetabolised benzene in exhaled air (breath), blood, and urine, as well as benzene metabolites in urine among occupationally exposed workers [17–24]. The other way is to use blood MetHb concentration to assess exposure and for risk assessment in order to provide information on the internal dose received by individuals [19–24]. In turn, this dose can be related to health outcomes.

In studying the acute toxicological effects of diesel and crude oil, that also contain benzene, in experimental animals an increase in dose of the fuel administered into the animals caused a dose dependent decrease in haemoglobin (Hb) and packed cell volume (PCV) [25, 26]. The observed linear reductions in haematological parameters demonstrated and suggested an anaemic condition in the animals.

Automobile Mechanics (AM) and Petrol Station Attendants (PSA) are commonly found in Nigeria. The AM does routine maintenance and repair of motor vehicles while the PSA dispenses PMS and other petrochemical products at automobile re-fueling stations. In Nigeria AM are commonly exposed to PMS by sucking with their mouth through a tube in an attempt to siphon PMS from the vehicle tank. They also often wash vehicle parts with PMS without any gloves. PSA practice dispensing the fuel into vehicles without using any protective device to minimize their exposure. In the process the AM and the PSA inhale the PMS fume. Therefore, automobile re-fueling and repairs are reasonable sources of benzene exposure among PSA and AM in Nigeria. This raises serious public health concern. These classes of workers are seldom subjected to pre-employment medical examination or provided with regular medical check ups to detect potential serious risk the exposure may have.

The purpose of the research was to investigate the potential risk of exposure to PMS fumes in Calabar, Nigeria, among AM, PSA and the general population based on the MetHb level as a blood biomarker of exposure to benzene in the PMS fume. Direct levels of benzene in blood and urine were not measured.

## 2. Materials and Methods

This was a population-based-cross-sectional study in which MetHb levels were estimated for individuals who were routinely exposed to PMS.

### 2.1. Sampling Procedure and Sample Size

The sample size for the study was 150 subjects. A sampling frame was drawn comprising all 368 automobile workshops in Calabar. Every 5th workshop was selected from this frame to make a total of 50 workshops. One workshop attendant was chosen per workshop by simple balloting provided he was 18 years and above for a total of fifty AM. The same procedure was carried out in selecting 50 PSA from 320 automobile fuelling stations. 

A total of fifty subjects were also selected from the general population (not targeted occupational groups) using the same criteria of age 18 years and above, whose work did not involve use of PMS and they were not working in automobile mechanic workshops or automobile fuelling stations. These 50 individuals represented this study's control subjects.

Informed consent was obtained for in-depth interview of the PSA and AM and collection of venous blood for PCV, and MetHb estimation. The PSA were asked about use of face mask and rubber-hooded delivery pump, and AM about sucking petrol from reservoirs or containers using their mouth. The duration of exposure was taken as the length of work in their various occupations.

### 2.2. Blood Collection, MetHb and PCV Estimation

Four millilitres of blood were collected by standard vene-puncture method from the 150 subjects. The blood was dispensed into EDTA bottle to give a concentration of 1–5 mg/mL. The samples were transported to the haematology laboratory of the University of Calabar Teaching Hospital, (UCTH) within one hour of collection, using field ice boxes for maintaining a temperature of between 18°C to 24°C. Samples were analysed within one hour of collection. Those that could not be analysed were diluted and stored in a refrigerator at 2°C to 4°C but not kept beyond 24 hours. Analysis was by method of Evelyn and Malloy [27] . About 0.2 mL of blood was lysed in a mixture of 4 mL of phosphate buffer (pH 6.8) and 6 mL of non-ionic detergent. The lysate was divided in equal volumes into tubes A and B. The absorbance of A was read with a spectrophotometer [28] at 630 nm and recorded as *D*_1_. Using a spectrophotometer the absorbance spectrum of MetHb exhibits a small characteristic peak of 630 nm. Addition of cyanide eliminates the peak and the decrease in absorbance is proportional to the MetHb concentration. Addition of ferricyanide measures total haemoglobin after conversion to MetHb. One drop of potassium cyanide was added to A, mixed and absorbance recorded as *D*_2_. One drop of potassium ferricyanide was added to B, mixed and allowed to stand for 5 minutes and absorbance recorded as *D*_3_. One drop of potassium cyanide was added to B, mixed and read as absorbance *D*_4_.

Result was expressed as MetHb (%) = (*D*_1_ − *D*_2_/*D*_3_ − *D*_4_) × 100. Four millilitres of phosphate buffer plus 6 mL non-tonic detergent was used as blank.

For the determination of packed cells volume [PCV] the method in Dacie and Lewis was adopted [29]. Anti coagulated blood was filled into plain capillary tube up to three quarters level by capillary suction. One end of the capillary tube was sealed using plasticin and the capillary was centrifuged using a special speed automated microhaematocrit centrifuge [30] at 1200 rpm for 5 minutes. PCV was read with micro haematocrit reader [31] and results were expressed in percentages and frequencies. The data were analysed using Epi-Info 2002 Epidemiological Software for Microcomputers [32]. Comparisons and associations were determined using Chi-square tests to determine significant difference at 95% degree of confidence (*P* < .05).

## 3. Results

The age range of subjects was between 18 and 56 years with a median age of 28 years for PSA, 27 years for AM, and 31 years for controls, Table 1. Twenty nine (58%) AM, 36 (72%) PSA and 25 (50%) were between 20–39 years old. Extending the age range to between 20 and 49 there were 39 (76%) AM, 42 (88%) PSA and 37 (74%) controls. 

**Figure 1:**
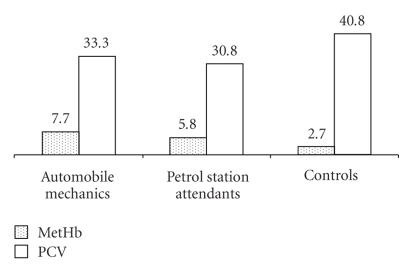
MetHb (%) and PCV (%) levels in Automobile Mechanics (AM), Petrol Station Attendants (PSA) and Controls.

Figure 1 shows that MetHb values were significantly higher (*P* < .05) in AM (7.7%) and PSA (5.8%) than in the control subjects (2.7%). Among the study subjects. 

Table 2 indicates that long *self-reported* exposure relates positively with the increased formation of MetHb and decreased PCV. The AM and PSA who had been exposed to the PMS fumes for three or more years and above had higher concentration of MetHb (7.2% to 13.6%) than those that were exposed for less than three years (1.8% to 2.5%). The measured PCV levels in the studied AM and PSA also decreased as the number of reported years of exposure increased.

Thirty two (64%) of the AM admitted sucking PMS while all of them (100%) had washed their hands with PMS (Table 3). The measured MetHb levels among those who reported they sucked PMS rose from 8.9% in those who sucked once or twice a day to 10.2% in those who sucked up to three times or more a day.

Those who washed their hands with PMS up to and above five times a day had a higher MetHb (13.6%) than those who washed their hands once or twice a day (10.6%).

The PCV levels among those who sucked three times daily were lower (30.4%) than those who sucked once a day (32.2%). Those who washed their hands up to and over five times a day had a lower PCV (28.8%) than those who washed their hands once a day.

Among the fifty PSA that were tested all (100%) denied ever using face mask and/or rubber-hooded dispensing pump. Four (8%) of them admitted ever going into the underground storage facility to measure the volume of PMS in the absence of a testing instrument. 

## 4. Discussion

In this study it was observed that AM and PSA had a significantly higher MetHb than the controls (*P* < .05). It was also seen that those exposed to the PMS fumes were having significantly lower PCV values compared with controls (*P* < .05). These findings agree with those of Dede and Kagbo [25], Ovuru and Ekweozor [26], Mohorovic [29] and Linz et al. [30]. The MetHb concentration as well as in AM were observed to be significantly higher than those of PSA (*P* < .05). This marked difference suggests that the AM are more exposed to the product since they suck PMS from the car tanks and use it for washing most of the engine parts. This agrees with the report that the practice of aspiration of PMS by mouth by mechanics was an important source for exposure [2, 31]. Although the primary route of exposure to benzene, a volatile chemical organic compound, is by inhalation of PMS fume, PMS came in contact with airways and lungs and through the skin when the study subjects either washed their hands and/or vehicle parts or took in some quantity of PMS.

Low dose exposure to volatile organics including benzene in PMS fume is proportional to age [10]. Over 70% of the AM (76%), PSA (88%) and Controls (74%) were of the same age range, therefore, the observed higher level of MetHb among the AM and PSA than the control was not due to age difference only.

Exposure of attendants to PMS fume has been limited in the USA by the use of rubber hood over the delivery pump and use of “self-service” at stations. Although a very small percentage admitted going into the underground storage to measure the volume of PMS the practice itself is extremely dangerous. The extreme heat generated in the underground facility produces more PMS fumes which have little escape route. It is therefore very hazardous to enter into such enclosure.

Comparing the years of exposure of both groups this study reveals that there is an increase formation of MetHb as the years of exposure increases. In Nigeria PMS at fuel stations is still largely dispensed by attendants who are exposed to the gasoline fumes for more than a typical 40-hour work week.

 The American Conference of Government and Industrial Hygienist (ACGIH) and National Institute of Occupational Safety and Health (NIOSH) recommend an occupational exposure limit of 23 mg/m^3^ for a 10-hour work day in a 40-hour work week [32].

## 5. Conclusion

This study has suggested increased exposure to PMS fume among AM and PSA. While the primary route of exposure to benzene is by inhalation of PMS fume benzene permeated the pulmonary and dermal exposure routes of the subjects when they either washed their hands and/or vehicle parts or took in some quantity of PMS. It has also shown that MetHb is a useful biomarker in determining the level of exposure to PMS fume. We recommend enlightenment campaign to educate AM and PSA on the negative health implication of exposure to the fume. We also recommend a legislation on mandatory provision of personal protection and monitors for AM and PSA, and phasing out of attendants at pumps adopting the use of self dispensing pump alternative. Finally we recommend the use of MetHb level during a mandatory medical examination for suitability or otherwise of AM and PSA for such employment or to continue in such employment.

## Figures and Tables

**Table 1:** Age distribution of study subjects.

Age range (years)	Automobile mechanics AM (%)	Petrol station attendants PSA (%)	Control
<20	5 (10)	4 (8)	6 (12)
20–29	18 (36)	23 (46)	10 (20)
30–39	11 (22)	13 (26)	15 (30)
40–49	10 (20)	8 (16)	12 (24)
≥50	6 (12)	2 (4)	7 (14)

Total	50 (100)	50 (100)	50 (100)

**Table 2:** Mean methaemoglobin (MetHb) and pack cell volume (PCV) values among Automobile Mechanics (AM) and Petrol Station Attendants (PSA) according to self-reported duration of exposure to Petrol (PMS) fumes.

		Duration of self-reported exposure to Petrol (PMS) fumes						
Automobile mechanics (AM)		<1 yr (*n* = 8)	1-2 yrs (*n* = 9)	3-4 yrs (*n* = 10)	5-6 yrs (*n* = 8)	7-8 yrs (*n* = 6)	9-10 yrs (*n* = 5)	>10 yrs (*n* = 4)
	MetHb (%)	2.1	2.6	8.7	9.1	10.1	11.7	15.0
	PCV (%)	41.8	41.4	32.5	30.3	30.0	28.6	24.4

Petrol station attendants (PSA)		<1 yr (*n* = 11)	1-2 yrs (*n* = 13)	3-4 yrs (*n* = 7)	5-6 yrs (*n* = 9)	7-8 yrs (*n* = 5)	9-10 yrs (*n* = 3)	>10 yrs (*n* = 2)
	MetHb (%)	1.8	2.5	7.2	7.8	8.7	10.8	13.6
	PCV (%)	40.4	40.2	35.0	35.0	32.0	30.0	28.0

**Table 3:** Mean methaemoglobin (MetHb) and pack cell volume (PCV) based on route and self-reported duration of exposure of AM to Petrol (PMS) fumes.

Route of exposure	Estimated (self-reported) daily frequency of exposure			
	<1 time	1-2 times	3-4 times	≥5 times

	Mean MetHb (%)			

Aspiration of petrol by mouth (*N* = 32)	0.0 (*n* = 0)	8.9 ± 2.7 (*n* = 23)	10.2 ± 2.6*(*n* = 9)	0.0 (*n* = 0)
Washing of hands with pPetrol (*N* = 50)	0.0 (*n* = 0)	10.6 ± 2.6 (*n* = 20)	11.1 ± 3.8 (*n* = 16)	13.6 ± 5.6*(*n* = 14)

	Mean pack cell volume (PCV)			

Aspiration of petrol by mouth (*N* = 32)	0.0 (*n* = 0)	31.4 ± 4.9 (*n* = 23)	30.4 ± 4.6 (*n* = 9)	0.0 (*n* = 0)
Washing of hands with petrol (*N* = 50)	0.0 (*n* = 0)	30.8 ± 4.8 (*n* = 20)	30.2 ± 4.6 (*n* = 16)	28.8 ± 3.8*(*n* = 14)

*P value <.05.
